# Electrical Conductivity of New Nanoparticle Enhanced Fluids: An Experimental Study

**DOI:** 10.3390/nano9091228

**Published:** 2019-08-29

**Authors:** Elena Ionela Chereches, Alina Adriana Minea

**Affiliations:** Faculty of Materials Science and Engineering, Technical University “Gh. Asachi” of Iasi, Bd. D. Mangeron no. 63, 700050 Iasi, Romania

**Keywords:** electrical conductivity, nanofluids, experimental, temperature variation

## Abstract

In this research, the electrical conductivity of simple and hybrid nanofluids containing Al_2_O_3_, TiO_2_ and SiO_2_ nanoparticles and water as the base fluid was experimentally studied at ambient temperature and with temperature variation in the range of 20–60 °C. A comparison of the experimental data with existing theoretical models demonstrated that the theoretical models under-predict the experimental data. Consequently, several correlations were developed for nanofluid electrical conductivity estimation in relation to temperature and volume concentration. The electrical conductivity of both simple and hybrid nanofluids increased linearly with both volume concentration and temperature upsurge. More precisely, by adding nanoparticles to water, the electrical conductivity increased from 11 times up to 58 times for both simple and hybrid nanofluids, with the maximum values being attained for the 3% volume concentration. Plus, a three-dimensional regression analysis was performed to correlate the electrical conductivity with temperature and volume fraction of the titania and silica nanofluids. The thermo-electrical conductivity ratio has been calculated based on electrical conductivity experimental results and previously determined thermal conductivity. Very low figures were noticed. Concluding, one may affirm that further experimental work is needed to completely elucidate the behavior of nanofluids in terms of electrical conductivity.

## 1. Introduction

Nanofluids are considered, at this point, a possible new generation of heat transfer fluids. Over the last few years, these new fluids have been studied both experimentally and numerically for different heat transfer applications [1,2,3]. Nevertheless, regardless of the tremendous work, nanofluid property estimation requires further systematic studies and, despite all the published research in this area, electrical conductivity is the least studied property compared with thermal conductivity or even viscosity and specific heat [4,5,6].

The electrical conductivity of nanofluids is associated with the capacity of charged nanoparticles in the fluid to transport the charges toward corresponding electrodes once an electric potential is applied [7,8]. Abdolbaqi et al. [9] discussed the relationship between thermal and electrical conductivity, which can be considered as an important parameter to assess the possibility of a specific nanofluid to be employed in an electrically active heat transfer application [9]. While the stability of a suspension depends on its electrostatic characteristics (for example, the zeta potential, which is very important in electrical conduction progression), electrical conductivity might also provide important information about nanofluid stability [8,9,10].

Zakaria et al. [11] discussed nanofluid adoption as an alternative coolant for a proton exchange membrane (PEM) fuel cell and noticed that a 0.1% alumina–water nanofluid gives very good results as an alternative fluid for PEM cells. Also, the same authors [12] introduced the thermo-electrical conductivity ratio (TEC) based on the ratio of the electrical and thermal conductivity of Al_2_O_3_ in water and water–ethylene glycol.

Cruz et al. [10] debated about alumina nanofluid property estimation and concluded that electrical conductivity can be a very good tool for exploring the nanoparticle structure in suspensions and that an external electric field can be used as a tool to design that particle structure. Further studies on alumina nanofluids were performed by Ganguly et al. [5] who measured the electrical conductivity of different concentrations of alumina–water nanofluids at different temperatures and concluded that the influence of temperature is much lower than the influence of concentration. In addition, Ganguly et al. [5] discussed the application of the Maxwell model for estimating the electrical conductivity of low dilution suspensions (less than 1% volume fraction) and found no agreement.

Abdolbaqi et al. [9] suspended alumina nanoparticles in a BioGlycol–water mixture and measured electrical conductivity. Their reported results were in contradiction with the literature consensus. Specifically, the electrical conductivity decreased with the increase in volume concentration, while TEC increased.

Zyla and Fal [13] performed experiments on the electrical conductivity of a silicon dioxide–glycol nanofluid and found that if the concentration of nanoparticles in a nanofluid is increasing, the electrical conductivity will increase linearly. The same linear increase in electrical conductivity was noticed in the literature [14,15,16] for ethylene glycol-based nanofluids. For example, higher electrical conductivity enhancement (about 1500% for 0.05 volume fraction at 25 °C) was presented by Sharifpur et al. [15].

A less studied nanofluid was investigated by Sundar et al. [8]. The authors measured electrical conductivity at low volume fractions of nanodiamond (ND)–Ni nanofluids (0.02%, 0.05% and 0.1%) between 24 °C to 65 °C. Their results indicated a very high upsurge in electrical conductivity (up to 1339.81%) with an increase in particle concentration for both water and ethylene glycol as base fluids.

Nurdin and Satriananda [17] investigated water-based maghemite (γ-Fe_2_O_3_) nanofluids and found an electrical conductivity enhancement of up to 160.49%.

Islam et al. [18] showed results on titania nanofluids, which were also found not to agree with the Maxwell model [19]; that is, actually under-predicting the values of electrical conductivity. A linear variation in electrical conductivity of titania nanofluids with particle fraction was detected by Lopez and Biswas [20] for low ionic strength and no substantial effect for higher ionic concentrations.

ZnO nanofluids were experimentally studied by Shen et al. [21], who concluded that the electrical conductivity is influenced by Brownian motion, agglomeration, and stability within the nanofluid.

Shoghl et al. [22] performed a complex experimental study on several nanofluids with 0.03% CuO, TiO_2_, MgO, MWCNT, Al_2_O_3_ or ZnO and concluded that all nanofluids have better electrical conductivity when compared with the base fluid.

As outlined before, electrical conductivity might be an important parameter as it is able to offer valuable information on the dispersion and stability of suspensions. While some research with similar objectives have been reported [1,2,3,4,5], a methodical study addressing this very important property was not identified in the open literature. The present experimental study tries to shed some light on the electrical conductivity of nanofluids. Most prior research has been directed to alumina nanofluids [5,6,7,9,10,11,12] and only very few of them considered other types of nanoparticles [8,13].

The main aim of this research was to investigate the effective electrical conductivity of water-based oxide simple and hybrid nanofluids. Two simple aqueous nanofluids (silica and titania) were manufactured, along with two hybrid nanofluids (alumina and silica; alumina and titania). Results will be discussed both at ambient temperature and with temperature increases and will be compared with two conductivity models available in the literature. Additionally, several correlations are proposed, based on volume fraction and temperature variation.

## 2. Theoretical Models

Over the years, a number of experimental and theoretical investigations were performed on the conductivity of suspensions. A comprehensive review on this subject was published in 1993 by Banisi et al. [23], who categorized the available models into four main categories: classical solutions, ordered arrangements of dispersed phase, approximations involving no empirical parameters, and relations involving empirical parameters [23]. For electrical or thermal conductivity estimation, Maxwell and Bruggeman correlations [19,24] are the most referenced [1,2,3,4,5,6,7,8,9,10,11,12,13,14,15,16,17,18,19,20,21,22,23]. The Maxwell model [19] is the most well-known equation and can be applied mostly to low volume concentrations of spherical nanoparticles. The Maxwell model calculates the electrical conductivity of the nanofluid (κ_nf_) as a function of the electrical conductivity of nanoparticles (κ_np_) and of the base fluid (κ_f_), also taking into account the particle volume fraction (φ) [19]:

(1) $$\frac{\kappa_{nf}}{\kappa_{f}}=1+\frac{3\left(\left(\frac{\kappa_{np}}{\kappa_{f}}\right)-1\right)\varphi }{\left(\frac{\kappa_{np}}{\kappa_{f}}\right)+2-\left(\left(\frac{\kappa_{np}}{\kappa_{f}}\right)-1\right)\varphi }$$

The Maxwell model is dependant on the conducting nature of the nanoparticles and Cruz et al. [10] proposed the following equations as a modification of the classical model:

(2) $$\left(i)\;\frac{\kappa_{nf}}{\kappa_{f}}=1-\frac{3}{2}\varphi ,\;\mathrm{for}\;\kappa_{\mathrm{np}}\;«\;\kappa_{f}\;(\mathrm{insulating}\;\mathrm{particles}\right)\;\left(\mathrm{ii})\;\frac{\kappa_{nf}}{\kappa_{f}}=1,\;\mathrm{for}\;\kappa_{\mathrm{np}}\;=\;\kappa_{f}\;(\mathrm{equal}\;\mathrm{conductivity}\right)\;\left(\mathrm{iii})\;\frac{\kappa_{nf}}{\kappa_{f}}=1+3\varphi ,\;\mathrm{for}\;\kappa_{\mathrm{np}}\;»\kappa_{f}\;(\mathrm{highly}\;\mathrm{conducting}\;\mathrm{particles}\right)$$

Cases (i)–(iii) show the theoretical effect, as predicted by Maxwell’s model, of the particle volume fraction on the relative conductivity (κ_nf_/κ_f_) for a constant value of conductivity ratio (κ_np_/κ_f_).

The Bruggeman model [24] can be expressed as:

(3) $$1-\varphi =\frac{\kappa_{np}-\kappa_{nf}}{\kappa_{np}-\kappa_{f}}{\left(\frac{\kappa_{f}}{\kappa_{nf}}\right)}^{1/3}$$

Both the Maxwell and Bruggeman models can be applied to estimate the electrical conductivity of nanofluids with nanoparticles made from Al_2_O_3_, Cu, CuO, TiO_2_, etc. [8]. Nevertheless, their applicability is debated in the open literature [6,7,8,10] and no straightforward conclusion is provided.

In regard to these observations, in this article, the authors will also compare their experimental results with the theoretical models outlined previously (see Equations (1), (2) and (3)) in order to determine their possible application in electrical conductivity estimation of titania and silica water-based nanofluids.

## 3. Experimental

Three types of nanoparticles (TiO_2_, Al_2_O_3_ and SiO_2_) were considered for preparing the samples in this experimental study. All single and hybrid nanofluids were manufactured by dilution from concentrated suspensions acquired from Alfa Aesar (Kandel, Germany) and no surfactant was inserted. The new fluids were sonicated for 30 min to ensure good stability. More information about hybrid and simple nanofluid preparation can be found in the author’s previous research [25].

Electrical conductivity was measured with an Edge^®^ Multiparameter HI 2030 (Hanna Instruments, Cluj, Romania) equipment with an integrated temperature sensor and large measurement area. Up to 500 mS/cm and 0.01 μS/cm resolution can be reached in the measured area. The temperature measurement range is between −20–120 °C with an accuracy of ±0.2 °C. The measurement accuracy at 25 °C is ± 1%. The experiment was performed at ambient pressure and the overall accuracy of the data was calculated as 3%. The temperature variation was assured by a heating bath at a controlled temperature.

The equipment was initially calibrated with a HI7031 solution with known electrical conductivity (1413 μS/cm at 25 °C). After the calibration, different samples were tested at ambient temperature and at different temperatures in the range of 20–60 °C. After each measurement, the electrode was carefully cleaned with distilled water and dried at ambient temperature. For each sample, we performed 3–5 tests and the recorded data was the average value.

## 4. Results and Discussion

Electrical conductivity results will be discussed for the SiO_2_ and TiO_2_ nanofluids in the range of volume concentration of 1–3% and five hybrid nanofluids, namely 0.5% alumina and 0.5% silica; 0.5% alumina and 1% silica; 0.5% alumina and 1.5% silica; 0.5% alumina and 1% titania; and 0.5% alumina and 1.5% titania.

All employed nanoparticles are insulators [6,18,22,26,27], with very low electrical conductivity compared to water, as outlined in Table 1.

In this particular situation, the Cruz et al. model can be used for insulating particles. Thus, Case (i) will be discussed in this section.

### 4.1. Experimental at Ambient Temperature

**Figure 1:**
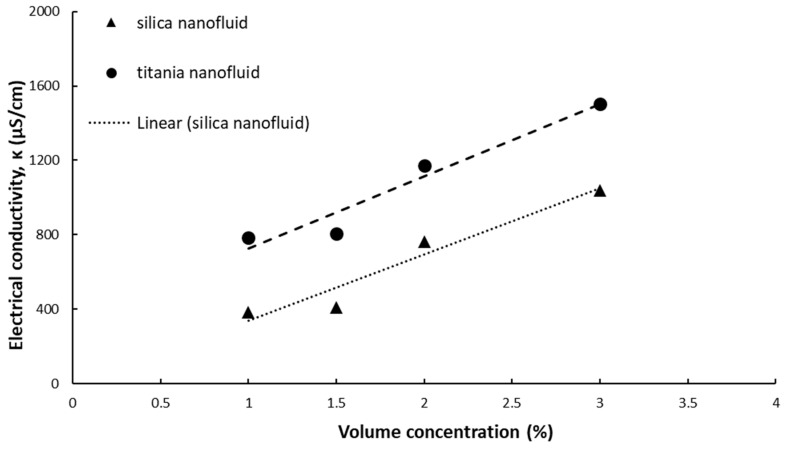
Experimental results on simple nanofluids at ambient temperature.

**Figure 2:**
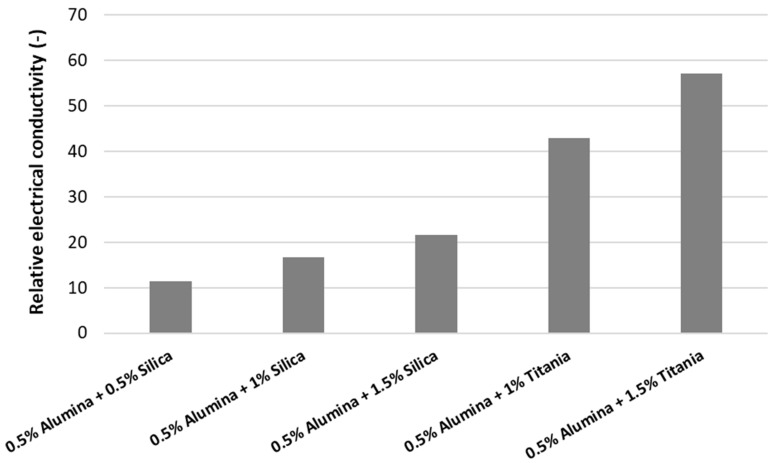
Experimental results on hybrid nanofluids at ambient temperature.

The experimental data of simple and hybrid nanofluids at ambient temperature are summarized in Figure 1 and Figure 2, while the electrical conductivity of water used for preparing the nanofluids was measured as κ = 25.77 μS/cm.

Electrical conductivity increases with volume concentration for both silica and titania simple nanofluids, as seen in Figure 1. The increase is linear and the correlations for electrical conductivity versus volume concentration can be expressed as:

For silica nanofluid:

κ = 354.57 φ - 16.57; R² = 0.94(4)

For titania nanofluid:

κ = 388.11 φ + 337.29; R² = 0.94(5)

where R^2^ is the R-squared value for each correlation.

When compared to water electrical conductivity, all nanofluids have a higher electrical conductivity. Silica nanofluids have a relative electrical conductivity of 14.82–40.28. Higher values were registered for titania nanofluids (30.38–50.28), mainly because the titania nanoparticle holds a higher electrical conductivity when compared to silica nanoparticles.

In Figure 2, one can notice that the electrical conductivity increases with increasing nanoparticle concentration. The titania and alumina nanofluids have higher electrical conductivity in comparison with alumina and silica hybrid nanofluids. This phenomenon occurs since the titania nanofluids have higher values for the electrical conductivity in regard to the silica nanofluid. Nevertheless, adding alumina to simple nanofluids seems to decrease the effective electrical conductivity of the hybrid nanofluid and this can be explained by the very low electrical conductivity of alumina nanoparticles and the possible lack of synergy between oxide nanoparticles.

In Figure 2, the relative electrical conductivity is defined as the ratio between the electrical conductivities of nanofluids and the base fluid: $\kappa_{r}=\frac{\kappa_{nf}}{\kappa_{f}}$.

Nevertheless, the variation in electrical conductivity may be due to the electrical double layer (EDL) or stability of the suspension. When nanoparticles are introduced in the base fluid (i.e., water), the EDL may appear and charge the particles. The particles may also transfer this charge to the dispersion, thus highly increasing the electrical conductivity of the nanofluid. Consequently, as the number of nanoparticles increases (corresponding to an upsurge in volume concentration), the effective electrical conductivity of the nanofluid increases. This author believes that the enormous enhancement of the nanofluid’s electrical conductivity is strictly dependent on two phenomena: one is the EDL formation around the nanoparticle surface inside the suspension and the second is the influence of the base liquid’s (i.e., water) polarity.

Similarly, Sarojini et al. [16] explained in their study with alumina nanoparticles that the high electrical conductivity augmentation can appear due to the formation of surface charges by the effect of nanoparticle polarization once distributed in water. The same explanation was also adopted by other authors (see Ganguly et al. [5] or Zakaria [11,12]).

Furthermore, the incidence of uniformly dispersed nanoparticles is considered. The decreased particulate masses determine an enlarged electrophoretic mobility, which upsurges the electrical conductivity of the nanofluid [5], even if the nanoparticles are insulators. If the nanoparticle volume concentration increases, the availability of conduction paths is higher in the suspension, which actually enhances the nanofluid electrical conductivity. Concluding, the higher the electrical conductivity, the better the nanofluid stability.

### 4.2. Experiments with Temperature Variation

**Figure 3:**
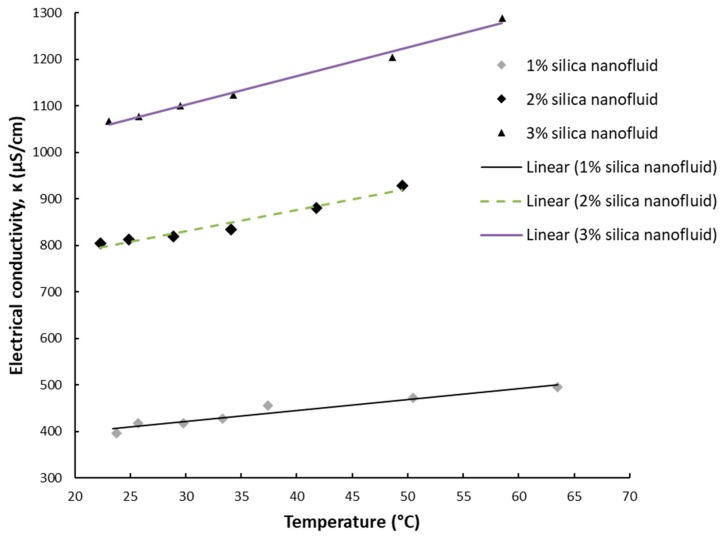
Experimental results on silica nanofluid electrical conductivity variation with temperature.

**Figure 4:**
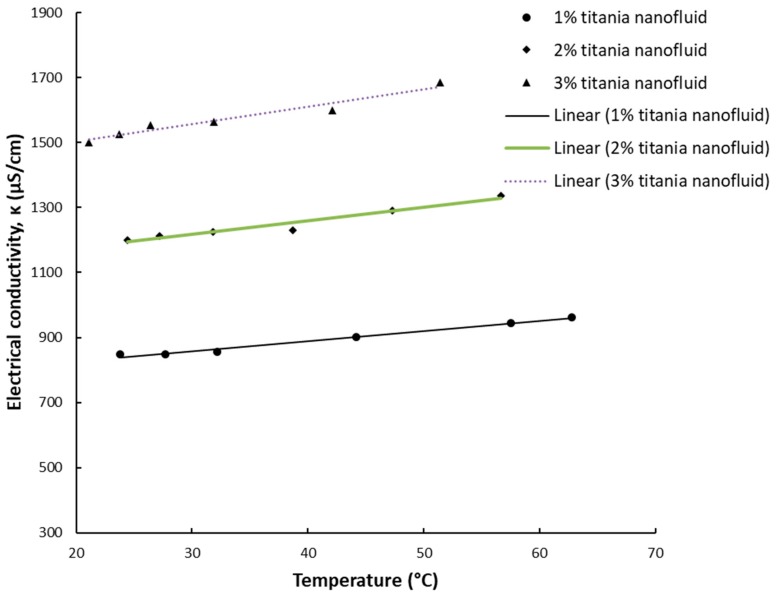
Experimental results on titania nanofluid electrical conductivity variation with temperature.

**Figure 5:**
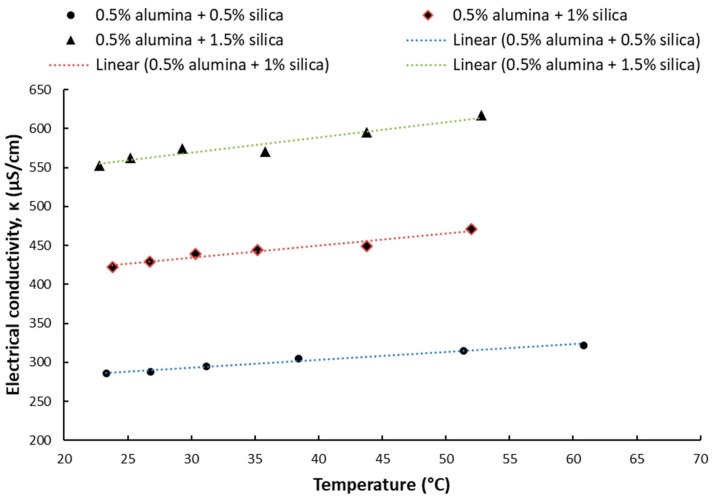
Experimental results on alumina + silica hybrid nanofluid electrical conductivity variation with temperature.

**Figure 6:**
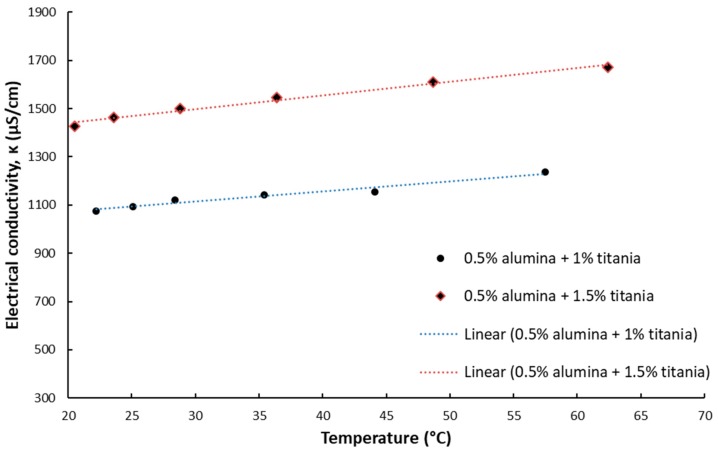
Experimental results on alumina + titania hybrid nanofluid electrical conductivity variation with temperature.

The results for electrical conductivity with temperature variation are depicted in Figure 3, Figure 4, Figure 5 and Figure 6 and one can notice that the conductivity increases linearly with temperature. Figure 3 and Figure 4 depict the experimental data for silica and titania simple nanofluids, while Figure 5 and Figure 6 contain the experimental outcomes for the alumina and silica and alumina and titania hybrid nanofluids.

Overall, the experimental work revealed that the electrical conductivity increases linearly with temperature with a percentage of 15–25% if compared to values at ambient temperature.

In connection with Figure 3, Figure 4, Figure 5 and Figure 6, the linear correlations are outlined in Table 2, along with the R-squared values for each equation.

The results are in line with most of the existing research [5,6,7,8,9], confirming that the electrical conductivity variation with temperature for water-based nanofluids have to be seen as linear, despite the nature of the nanoparticles suspended in water.

**Figure 7:**
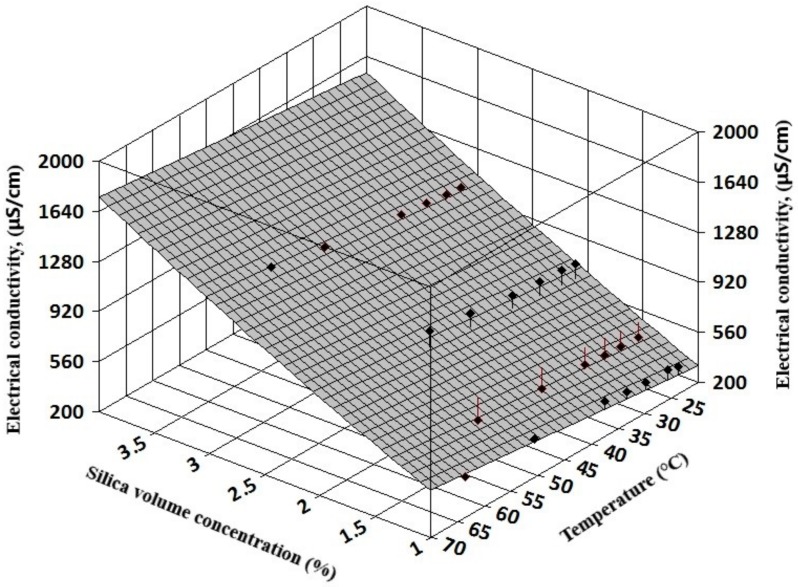
Three-dimensional analysis on silica nanofluids.

**Figure 8:**
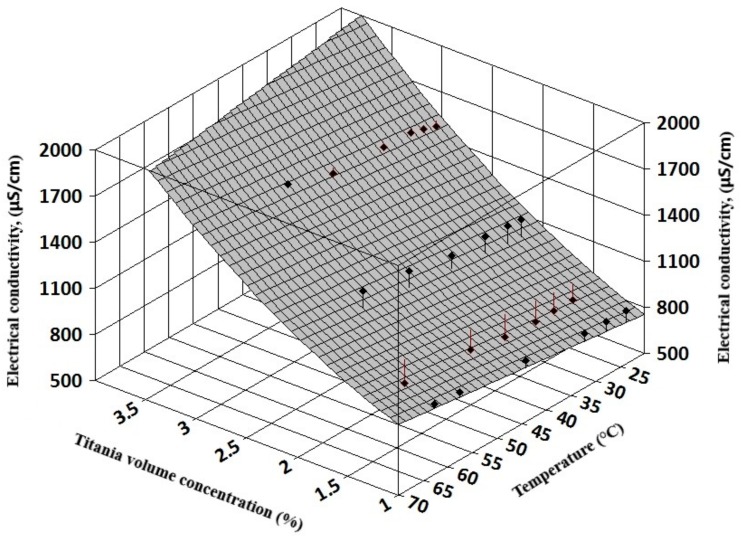
Three-dimensional analysis on titania nanofluids.

**Figure 9:**
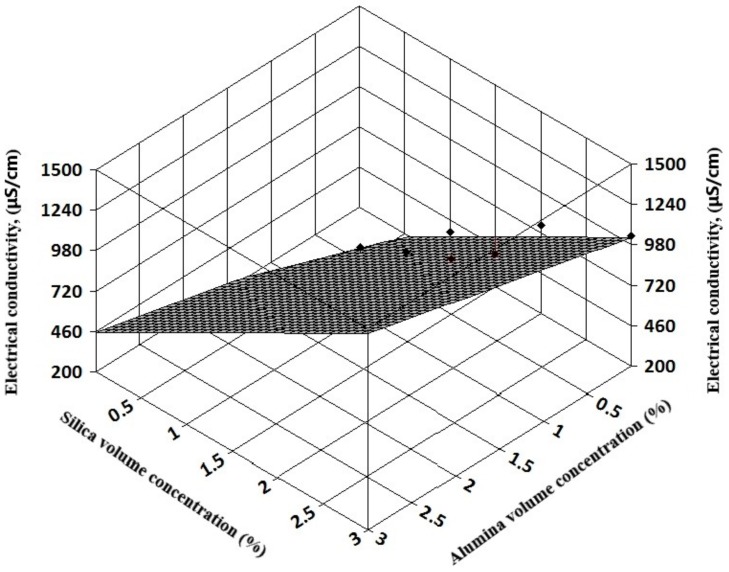
Three-dimensional analysis on alumina + silica hybrid nanofluids.

**Figure 10:**
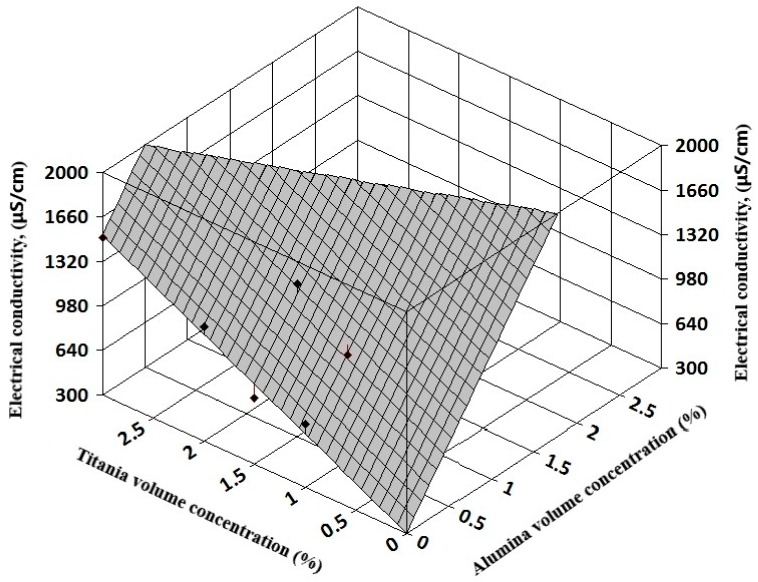
Three-dimensional analysis on alumina + titania hybrid nanofluids.

The complexity of experimental results allows a three-dimensional (3D) regression analysis that can offer information on electrical conductivity variation with both volume concentration and temperature for simple nanofluids (see Figure 7 and Figure 8). Also, for hybrid nanofluids, the 3D analysis can reveal the influence of each nanoparticle type on the hybrid nanofluid electrical conductivity (see Figure 9 and Figure 10).

The resulting regression equations that are valid for 1–3% nanoparticle volume concentration up to 60 °C are:

For silica nanofluids:

κ_nf_ = −103.47 + 315.14 φ + 17.23 φ^2^+ 4.45 T; R² = 0.94 (6)

For titania nanofluids:

κ_nf_ = 491.56 + 104.67 φ + 71.37 φ^2^+ 4.19 T; R² = 0.93 (7)

The resulting regression equations that are valid for 1–3% nanoparticle volume concentration at ambient temperature are:

For alumina + silica nanofluids:

κ_nf_ = 15.58 + 146.39 φ_1_ + 337.42 φ_2_; R² = 0.96 (8)

For alumina + titania nanofluids:

κ_nf_ = 302.03 + 956.65 φ_1_ + 406.92 φ_2_; R² = 0.95 (9)

In Equations (8) and (9), φ_1_ refers to alumina volume concentration and φ_2_ to silica and titania volume concentration, respectively.

### 4.3. Comparison with Analytical Models

**Figure 11:**
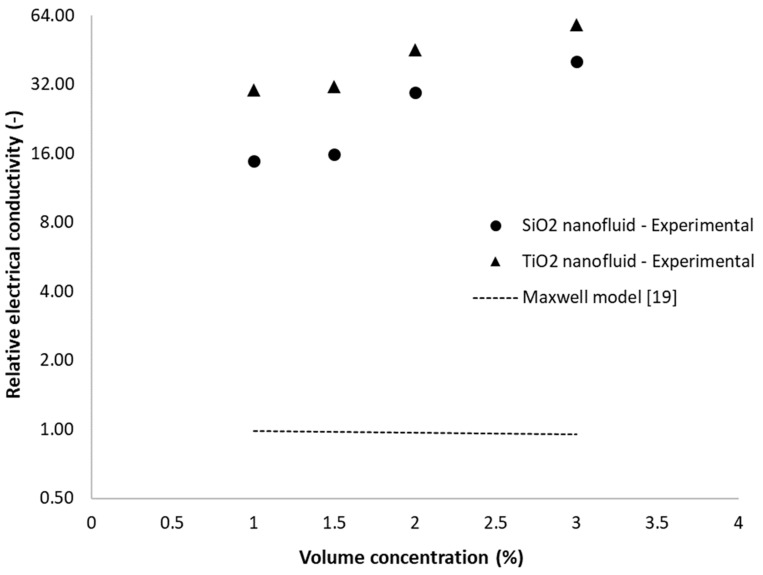
Comparison of experimental data with the Maxwell theoretical model.

As pointed out earlier, there are several theoretical models for conductivity estimation. Furthermore, in Figure 11 the experimental results versus one of the most used analytical equations, the Maxwell equation [19], are plotted. The Cruz et al. equation [10] and Brugemann model [24] give similar results to that of the Maxwell model, as shown in Table 3 (Equations (1)–(3)). The results for titania nanofluids are analogous to those obtained for the silica nanofluid (i.e., when three decimals are used, the results are identical), since both titania and silica nanoparticles have extremely low electrical conductivity in comparison with the base fluid (i.e., water). Thus, κ_np_ « κ_f_ and $\frac{\kappa_{np}}{\kappa_{f}}\to 0$. Consequently, in Figure 11, the dotted line exemplifies the Maxwell model values for both nanofluids.

One can see in Figure 11 and Table 3 that all three classical models are highly under-predicting the experimental values. All theoretical models predict a slight reduction in electrical conductivity while adding nanoparticles to the base fluid, a fact that is highly contradicted by the experimental study. This fact was noticed by almost all researchers as far as the authors are aware and was discussed in Section 2. This phenomenon appears to be due to the circumstance that, despite the physical properties of the nanoparticles and the base fluid, the electrical conductivity of nanoparticle suspensions (or even colloidal suspensions) reveals a more complicated dependency on many factors and not only on the electric layer. Another important aspect to be considered is the electrical synergy between nanoparticles in hybrid nanofluids as well as the synergy between the nanoparticle and the base polar liquid. Some phenomena may also be acknowledged as the modification in configuration of surface charges by the nanoparticle’s polarization once dispersed in water (i.e., also specifically if a polar fluid is considered). Other influencing factors may be acknowledged as: volume fraction, nanoparticle type, nanoparticle size, suspension stability and the presence of surfactant (if used). All these aspects are extremely hard to consider in a theoretical model and a lot of research has to be performed.

Based on all these remarks, the Maxwell, Cruz or Bruggeman models cannot correctly predict the augmentation in electrical conductivity and further equations have to be established (as proposed in this article).

### 4.4. Thermo-Electrical Conductivity Evaluation

As outlined in the introduction, a possible application of nanofluids are in proton exchange membrane fuel cells [11,12]. A parameter, TEC, was proposed in the literature to assess the possible use of a nanofluid in a fuel cell. The TEC expression is given as [12]:

(10) $$TEC=\frac{5\kappa_{nf}}{\kappa_{nf}\kappa_{f}}$$

Zakaria et al. [11,12] demonstrated that the higher the TEC value, the more advantageous the nanofluid is for fuel cell applications. Based on our calculations, which also relied on previous thermal conductivity experimental results [28], the TEC values are very low and decrease with the increase in concentration (Table 4).

As an observation, the 0.5% alumina + 0.5% silica hybrid nanofluid has the best TEC results as shown in Table 4, which is also based on thermal conductivity enhancement. Nevertheless, none of these nanofluids can be considered efficient for fuel cells.

## 5. Conclusions

Nanofluids are of great interest when it comes to new heat transfer fluids. However, even if thermal conductivity and viscosity are widely discussed in the archived literature, studies on electrical conductivity are rare.

Electrical conductivity was experimentally studied for several simple and hybrid nanofluids. The tests were performed in the range of 1–3% nanoparticle volume concentration dispersed in water at ambient temperature and for temperatures up to 60 °C. The complexity of the performed tests allowed an intricate analysis in regard to electrical conductivity dependence on nanoparticle type, volume concentration and temperature.

The results were in line with current published observations and showed a very large increase in electrical conductivity when oxide nanoparticles are added to water. In addition, the electrical conductivity linearly increases with volume concentration and with temperature. The relative values are up to almost 60 times higher than that of the base fluid (which was water for this particular study). More precisely, for silica nanofluids, the enhancement in electrical conductivity goes from 14 times to 40 times, while for titania nanofluids, the increase goes from 30 times to 58 times higher than the base fluid. In regard to hybrid nanofluids, the augmentation in electrical conductivity is higher for the alumina–titania hybrid nanofluid, going from 43 times to 57 times higher when compared to water.

These experimental tests allowed the authors to propose several correlations for the estimation of electrical conductivity, as well as multi-parameter correlations based on both volume fraction and temperature.

The TEC parameter was found to be very low. However, the 0.5% alumina + 0.5% silica hybrid nanofluid could be a good option for fuel cells when compared to simple oxide nanofluids.

As an overall conclusion, electrical conductivity is an important parameter and can be considered as a measure of nanofluid stability, however it strongly affirms that further experimental research is needed on this extremely poorly studied parameter.

## Figures and Tables

**Table 1:** Electrical conductivity of nanoparticles.

Nanoparticle Type	Electrical Conductivity, μS/cm	Reference
Alumina	10^−8^	[6]
Silica	10^−9^	[26]
Titania	10^−2^	[22,27]

**Table 2:** Correlations for electrical conductivity variation with temperature.

Nanofluid Type and Concentration	Correlation	R-Squared Value
1.0% silica	K = 2.35 T + 351.33	R² = 0.94
2.0% silica	K = 4.57 T + 692.92	R² = 0.96
3.0% silica	K = 6.13 T + 918.56	R² = 0.99
1.0% titania	K = 3.11 T + 764.61	R² = 0.99
2.0% titania	K = 4.16 T + 1091.8	R² = 0.95
3.0% titania	K = 5.38 T + 1395.00	R² = 0.96
0.5% alumina + 0.5% silica	K = 0.98 T + 263.65	R² = 0.99
0.5% alumina + 1.0% silica	K = 1.54 T + 387.76	R² = 0.95
0.5% alumina + 1.5% silica	K = 1.94 T + 511.02	R² = 0.94
0.5% alumina + 1.0% titania	K = 4.18 T + 989.37	R² = 0.96
0.5% alumina + 1.5% titania	K = 5.674 T + 1327.6	R² = 0.99

**Table 3:** Estimation of relative electrical conductivity using the Brugemann and Cruz models versus the Maxwell model for silica–water nanofluids.

Nanofluid Type and Concentration	Maxwell Model [19]	Brugemann Model [24]	Cruz et al. Model [10]
1.0% silica	0.985	0.985	0.985
1.5% silica	0.977	0.978	0.978
2.0% silica	0.970	0.970	0.970
3.0% silica	0.956	0.955	0.955

**Table 4:** Thermo-electrical conductivity ratio (TEC) values for studied simple and hybrid nanofluids.

Nanofluid Type and Concentration	TEC
1% silica	0.015
1.5% silica	0.014
2% silica	0.008
3% silica	0.006
1% titania	0.007
1.5% titania	0.007
2% titania	0.005
3% titania	0.004
0.5% alumina + 0.5% silica	0.019
0.5% alumina + 1% silica	0.013
0.5% alumina + 1.5% silica	0.010
0.5% alumina + 1% titania	0.005
0.5% alumina + 1.5% titania	0.004

## Nomenclature

κ	thermal conductivity, W/m °C
T	temperature, °C
R^2^	accuracy of linear correlations, -
κ	electrical conductivity of the nanofluid, µS/cm
ϕ	particle volume concentration, %
φ	particle volume fraction, -
*Subscripts*
f	refers to base fluid
nf	refers to nanofluid
np	refers to nanoparticles
r	refers to relative
*Abbreviations*
EDL	electrical double layer
TEC	thermo-electrical conductivity ratio
PEM	proton exchange membrane
